# Commentary: Pattern destabilization and emotional processing in cognitive therapy for personality disorders

**DOI:** 10.3389/fpsyg.2018.01845

**Published:** 2018-10-09

**Authors:** Lois A. Gelfand, Michaela C. Ervin, Sophie R. Germ

**Affiliations:** Department of Psychology, University of Pennsylvania, Philadelphia, PA, United States

**Keywords:** dynamical systems theory, catastrophe theory, clinical psychology, psychotherapy, treatment outcome, treatment process

Hayes and Yasinski (2015) analyze negative and positive functioning in personality disorder (PD) patients undergoing cognitive therapy. Noting that psychotherapeutic change is often “not gradual and linear” (Hayes and Yasinski, 2015, p. 2) they focus on destabilization as a predictor of outcome. The authors connect their work to dynamical (or dynamic, Hayes and Strauss, 1998) systems theory (DST), a framework from mathematics and physics, stating that their findings “are consistent with…principles from dynamic systems theory,” (Hayes and Yasinski, 2015, p. 1). They use DST concepts, terminology, and research to explain their hypotheses and results. They also state that they have not conducted “true dynamic systems analysis and modeling” (Hayes and Yasinski, 2015, p. 11).

Because this article is still widely read and cited (see http://loop-impact.frontiersin.org/impact/article/120115#totalviews/views), we believe the authors should clarify whether they use DST terms literally or metaphorically. Literally means that DST approaches model psychological change, or that the underlying processes are a dynamical system. Evidence of these could justifiably motivate clinical researchers to pursue applications of DST. Metaphors suggest that two concepts are similar in limited, albeit vivid, ways, but fundamentally different, and do not raise expectations of literal applications in the future.

Because DST is unfamiliar to most psychologists, readers may have difficulty distinguishing metaphorical from literal usage; readers might mistakenly conclude from metaphors that functional states are known to be *attractors*, or that we are close to proof that destabilization must precede psychotherapeutic change.

We try to fit the authors' concepts to literal interpretations of DST terminology, to clarify their relationship. The dynamical system (DS) in DST is a set of *state variables* whose values change over time according to deterministic functions collectively called the *time evolution law (TEL*; Katok and Hasselblatt, 1995), a set of feedback equations with constants called *control variables*. Knowing the *state variables'* initial values, the *control variable* values, and absent outside influences, we can know the system's state at any future time, and can follow the *state variable* trajectories between any two time points. Outside influences include *perturbations*, which change the values of the *state variables*, bumping the system into a new state, from which it then evolves. For some TEL equations and *control variable* values, *state variables* can evolve into an *attractor*, one or more values where neighboring trajectories of *state variables* with different starting values converge. *Attractor* types include point (a single value), periodic (an oscillating pattern), and chaotic, which is complicated but may account for some adaptive biological phenomena (see Wagner and Persson, 1998). Hayes and Yasinski (2015, p. 2–3) state, “patterns of (PDs) can be conceptualized as attractors” and “Dozois et al. (2009)…suggest that the development of…a new attractor might account in part for the prophylactic effects of cognitive therapy.”

Hayes and Yasinski (2015, p. 1) describe PD patterns as “entrenched,” perhaps conceptualizing them as *point attractors*, and characterize mentally healthy states as “flexible and adaptive.” We wonder if they conceptualize these as stable positive *point attractors* or *chaotic attractors*, and if there is evidence that negative and positive pattern activation (the presumed *state variables*) form either type of *attractor*.

Hayes and Yasinski (2015, p. 9) invoke random DST when they suggest that something “akin to…flickering” (*state variables* demonstrating occasional jumps between alternative *attractors*; Dakos et al., 2013) might occur in psychotherapeutic transitions. Flickering occurs in random DSs, where the deterministic TEL is combined with a noise variable representing random *perturbations* of *state variables*; a literal reference requires random *perturbations*, which the authors did not discuss.

Hayes and Yasinski (2015) also invoke catastrophe theory (CAT), families of mathematical functions that can model discontinuous system transitions (termed catastrophes). CAT models depend on *control variables* (Gilmore, 1992); In a DS, as the *control variables* gradually change, one *attractor* disappears and a new *attractor* appears. While initially, the *state variables* also change gradually, they eventually make a sudden switch from the old to the new *attractor*. Hayes and Yasinski (2015, p. 2) present dispersion as a measure of an “early [indicator] of system transition”—a CAT concept also known as a “diagnostic catastrophe flag” (Gilmore, 1992, p. 86), a change in system behavior mathematically determined to occur prior to a catastrophe. Hayes and Yasinski (2015, p. 2) focus on the flag of “increased variability in system behavior” which they also refer to as “critical instability” and “destabilization.”

However, we could not identify potential *control variables*. Psychotherapy, which might represent a *control variable*, increasing in intensity over time, leading to psychotherapeutic change, is “conceptualized as a perturbation” (A random *perturbation*, justifying the flickering reference?) Without *control variables*, patients' functional states would not undergo discontinuous transitions related to CAT, and there would be no early indicator of destabilization to be measured by the dispersion variable.

Hayes and Yasinski (2015, p. 1) state that “Effective psychotherapy can be viewed as a way to perturb self-perpetuating and disabling patterns to facilitate new learning and more adaptive functioning,” suggesting that *perturbations* alone can change a DS *attractor* state. This can indeed occur if a large enough *perturbation* knocks *state variables* close enough to a different *attractor*, but only in a DS with more than one *attractor* for the same *control variable* values. This type of *attractor* transition is not the kind of discontinuous change addressed by CAT models.

Hayes and Yasinski (2015) postulate “two types of variability: (1) *opening and loosening…*. and (2) *destabilizing.”* (Hayes and Yasinski, 2015, p. 3). “Destabilizing” references the diagnostic catastrophe flag discussed above; we wonder what CAT/DST concepts correspond to “opening and loosening” variability. We also wonder what it means to “activate …. attractors” (Hayes and Yasinski, 2015, p. 2).

If the authors use DST /CAT terms literally, much work remains to make a convincing case (see Gelo and Salvatore, 2016; Schiepek et al., 2018, for alternative DST approaches). If metaphorically, they should state that the terms are intended to inspire novel research approaches, but not to imply a literal relationship to DST. We ask the authors to clarify the relationship between DST and this work.

## Author contributions

LG primary responsibility for conception, design, interpretation. All authors contributed to manuscript revision, read, and approved the submitted version.

### Conflict of interest statement

The authors declare that the research was conducted in the absence of any commercial or financial relationships that could be construed as a potential conflict of interest.
